# Supplementary material for: Evaluating route preview as an alternative to turn-by-turn navigation in pedestrian mobility
Source: PLoS One. 2026 Mar 23;21(3):e0340711. doi: 10.1371/journal.pone.0340711 (PMC13008247; doi:10.1371/journal.pone.0340711)
Supplement: Attachment — Submitted filename: Response to ReviewersSchoeningPlos.pdf [file pone.0340711.s003.pdf]

University of St.Gallen

Dufourstrasse 50, 9000 St.Gallen

University of St.Gallen
Institut for Computer Science
Torstrasse 25
9000 St. Gallen

Prof. Dr. Johannes Schöning
Chair of Human-Computer Interaction
T +41 71 224 3331
johannes.schoening@unisg.ch
unisg.ch

St.Gallen, 4 Novemer 2025

Response to Reviewers for Manuscript PONE-D-25-10260, "Evaluating Route Preview as an Alternative to Turn-By-Turn Navigation in Pedestrian Mobility"

Dear Dr Sehra, dear Ms Wagan, dear Editing Team,

dear Madam / Dear Sir

Please find attached our revision report in this document. I have attached the other editorial request at the end of this PDF as well, so the process is clearly documented.

We thank you and the reviewers for the insightful feedback and constructive criticism on our manuscript, "Evaluating Route Preview as an Alternative to Turn-By-Turn Navigation in Pedestrian Mobility" (PONE-D-25-10260). We appreciate the time and expertise you have dedicated to this review process. We have thoroughly revised the manuscript, taking every comment into careful consideration, and believe the paper is now substantially improved and suitable for publication in PLOS ONE.

We appreciate your editorial guidance. This feedback was pivotal, pushing us to articulate explicit research questions that now provide a robust theoretical scaffold for our three-study design. Below, we provide a point-by-point response to the journal requirements and the specific concerns raised by each reviewer, detailing the changes made in the revised manuscript

Response to Journal Requirements

We have carefully reviewed all journal-specific requirements to ensure the revised manuscript is fully compliant with PLOS ONE's formatting and publication standards. Below, we detail the actions taken for each requirement.

1. PLOS ONE Style Requirements: We have reviewed our manuscript against the provided PLOS ONE style templates and have updated the formatting, file naming, and overall structure to meet the

journal's requirements. We are using the PLOS template version 3.6 Aug, August 2022, provided directly by Overleaf.

2. Inclusivity in Global Research Questionnaire: As requested, we have completed the PLOS questionnaire on inclusivity in global research and have uploaded the document as a Supporting Information file with our resubmission.

3. Financial Disclosure: We have updated our financial disclosure as requested. The statement now reads: "GS and JS - Swiss National Science Foundation (SNSF) grant number 200021 207430. The funders had no role in study design, data collection and analysis, decision to publish, or preparation of the manuscript."

4. Ethics Statement: The full ethics statement has now been integrated into the manuscript at the end of the Literature Review section (Section 2, page 6). The statement includes the full name of the approving committee ("Ethics Committee of the University of Monash") and the relevant project details ("Project Title: Exploring Navigation Skills using different methods, Project ID: 23126").

5. Copyrighted Map Images: We have addressed the issue of potentially copyrighted map images in the original Figures 1 and 4. The revised manuscript now only includes new, fully compliant illustrations. Therefore, we opted to remove Figure 1.

6. Figure 3 Reference: We have carefully checked all in-text figure references throughout the manuscript. All placeholder references (e.g., "???") have been replaced with the correct figure numbers, and we confirm that Figure 3 is now correctly referenced in the text.

7. Supporting Information Captions: Captions for all Supporting Information files have been added at the end of the manuscript, as per the journal's guidelines.

8. Identifying Data in Supporting Information: We have reviewed all Supporting Information files to ensure participant privacy. All potentially identifying data has been removed or fully anonymised. Please let us know if there is anything critical in the data set.

9. Consent for Interview Transcripts: We would like to clarify that our research did not involve the collection of interview transcripts. Study 3 was a design studio workshop, not a series of interviews. In accordance with our ethics approval, all participants provided written informed consent for their contributions (including anonymised quotes and design sketches) to be used for research purposes and publication.

Reviewer 1

We are grateful to Reviewer #1 for their valuable and constructive feedback. These comments have significantly improved the manuscript's structure, clarity, and engagement with the current state of the literature. Below, we address each point in detail.

1. References: We agree with the reviewer that the literature review needed updating. We have substantially revised the Literature Review (Section 2) to incorporate more recent and relevant studies, ensuring the manuscript reflects the current state of the field. As examples, we have added new citations

such as Dugas et al. [5] and Mazurkiewicz et al. [6] that discuss contemporary challenges in pedestrian navigation.

2. Submission Readiness: We apologise for the formatting errors in the initial submission.

3. Unsupported Claims: We thank the reviewer for highlighting areas where our claims required stronger support.

◦ 3a. (Page 2 Claim) The claim that turn-by-turn (TBT) instructions are not the best navigation option for pedestrians has now been substantiated with multiple citations. In the revised manuscript (page 2), this statement is now supported by references [1, 5, 6, 12, 13].

◦ 3b. (Page 3 Claim) The statement regarding the route previews (RP) potential to outperform TBT has been rephrased to be more nuanced. It is now presented as a potential avenue for future design improvements that our study explores, rather than an unsubstantiated claim (page 3).

◦ 3c. (Contradictory Claims) We have revised the text to remove any contradictions. The summary in Section 3.2.4 (page 9) now accurately reflects the quantitative data presented in the results (a 43.7% preference for RP), stating that a "considerable share" of participants preferred RP, which avoids any overstatement.

4. Article Structure in Introduction: A new paragraph has been added at the end of the Introduction (page 3) that explicitly outlines the structure of the article, guiding the reader through the subsequent sections.

5. Section 2 Title: The title of Section 2 has been changed from "Related Works" to "Literature Review" to reflect its content more accurately. The introductory text of the section has also been revised accordingly.

6. Survey Number in Table Captions: We have updated all relevant table captions to include the corresponding study number for improved clarity (e.g., Table 1, Table 2, and Table 3).

7. Small Sample Size in Study 3: We acknowledge the reviewer's concern about the small sample size in Study 3. The Introduction to Study 3 (Section 5, page 15) has been revised to better clarify its qualitative, exploratory nature as a design studio workshop. Furthermore, we have added a paragraph to the Discussion (page 20) that explicitly acknowledges the implications of this small sample size as a limitation of the study.

We believe these changes have greatly strengthened the manuscript and thank the reviewer again for their guidance.

Reviewer 2

We are particularly grateful to Reviewer #2 for their incisive critique. These comments challenged us to re-evaluate the manuscript's core narrative, prompting a significant reframing that has substantially elevated its scientific contribution

1. Lack of Clear Research Questions: We agree that research questions help to better guide readers. We have formulated three explicit Research Questions (RQ1, RQ2, RQ3), which are now presented in the

Introduction (page 2). These questions frame the entire study by clarifying the specific literature gaps we aim to address concerning pedestrian preferences, objective performance, and design enhancements for navigation aids.

2. Justification for Three Studies: The newly introduced Research Questions provide a clear and logical justification for the three-stage study design. The manuscript now explicitly links each study to a specific research question, creating a cohesive narrative:

◦ Study 1 addresses RQ1 by investigating the stated preferences of pedestrians.

◦ Study 2 addresses RQ2 by evaluating the objective performance of the two navigation modes.

◦ Study 3 addresses RQ3 by exploring potential design enhancements for RP.

3. Flawed Design of Study #1: We acknowledge the inherent limitations of using online surveys for assessing contextual preferences. In the revised manuscript, we have added a new paragraph in Section 3 (page 6) that explicitly recognises this limitation. The text now frames Study 1 as an essential first step that provides a baseline understanding of user attitudes, which is then tested against observed behaviour in Study 2.

4. Design of Study #2: We have added a comprehensive new paragraph in the methodology section for Study 2 (Section 4.1.1, page 10) to justify our design choices more robustly. This section now explains:

◦ The rationale for comparing TBT and RP on spatial learning tasks, which reflects a realistic user need to recall routes even when using a navigation aid.

◦ The controls we implemented to manage participant familiarity with the environment. This includes the recruitment of newly enrolled students and the use of familiarity as a covariate in our regression analysis, which confirmed its expected effect (Table 3).

◦ How these additions provide a more rigorous and balanced methodological framework for the field study, strengthening the validity of our findings. These additions not only resolve the reviewer's specific methodological concerns but also provide a more rigorous framework that strengthens our central—and perhaps surprising—finding: that a feature-limited RP mode performs on par with a fully-featured TBT system, even under more stringently controlled conditions.

5. Argument for Study #3: The Introduction to Study 3 (Section 5, page 15) has been completely rewritten. It is now clearly framed as the logical next step that directly follows from the findings of Studies 1 and 2. It addresses the key design question that emerges from our results: how could RP be improved to outperform TBT potentially?

We are confident that these substantial revisions have addressed the reviewer's critical concerns and have resulted in a significantly stronger and more coherent manuscript. Thank you once again for your time and effort.

Reviewer 3

We thank Reviewer #3 for their careful reading and helpful suggestions, which have improved the manuscript's professionalism and readability.

1. Date Formatting: We have corrected the date formatting in Section 5.1. The date is now spelt out as "June 12, 2024" (page 15) to prevent any ambiguity.

2. Figure Link Placeholders: The entire manuscript has been reviewed, and all placeholder figure references have been corrected with the proper figure numbers.

3. Citation Formatting: The reference list has been carefully checked, and the formatting of all citations has been standardised for consistency throughout the list.

We once again thank the editor and all reviewers for the time and effort invested in providing this constructive feedback. We are confident that the extensively revised manuscript is now a much stronger scientific contribution and is suitable for publication in PLOS ONE. We look forward to your evaluation of the revised submission.

Kind regards,

Gian-Luca Savino, Emanuel de Bellis, Reuben Kirkham, Johannes Schöning (Corresponding Author)

University of St.Gallen

Johannes Schöning
Chair of Human-Computer Interaction

Enclosures:
– Revision Letter v2 from October 2025

RE: Response to Reviewers for Manuscript PONE-D-25-10260, "Evaluating Route Preview as an Alternative to Turn-By-Turn Navigation in Pedestrian Mobility"

Subject: Re: PONE-D-25-10260R1 – Evaluating Route Preview as an Alternative to Turn-By-Turn Navigation in Pedestrian Mobility

Dear Ms Garcia Wagan,

Thank you very much for your message. It would be great if you could update the statement in the online submission form on our behalf, as we are unable to change it ourselves. The correct statement is:

"The funders had no role in study design, data collection and analysis, decision to publish, or preparation of the manuscript."

We have now included a dedicated Method and Ethics section before all three studies, labelled as Section 3.1, where we refer to the points you mentioned. We have also uploaded a new version of the manuscript accordingly.

Thank you very much for your support.

Kind Regards,
Johannes Schöning

Dear Dr Sehra,

We thank you and the reviewers for the insightful feedback and constructive criticism on our manuscript, "Evaluating Route Preview as an Alternative to Turn-By-Turn Navigation in Pedestrian Mobility" (PONE-D-25-10260). We appreciate the time and expertise you have dedicated to this review process. We have thoroughly revised the manuscript, taking every comment into careful consideration, and believe the paper is now substantially improved and suitable for publication in *PLOS ONE*.

We appreciate your editorial guidance. This feedback was pivotal, pushing us to articulate explicit research questions that now provide a robust theoretical scaffold for our three-study design.

Below, we provide a point-by-point response to the journal requirements and the specific concerns raised by each reviewer, detailing the changes made in the revised manuscript

Part 1: Response to Journal Requirements
We have carefully reviewed all journal-specific requirements to ensure the revised manuscript is fully compliant with PLOS ONE's formatting and publication standards. Below, we detail the actions taken for each requirement.

1. **PLOS ONE Style Requirements:** We have reviewed our manuscript against the provided PLOS ONE style templates and have updated the formatting, file naming, and overall

structure to meet the journal's requirements. We are using the PLOS template version 3.6 Aug, August 2022, provided directly by Overleaf.

2. **Inclusivity in Global Research Questionnaire:** As requested, we have completed the PLOS questionnaire on inclusivity in global research and have uploaded the document as a Supporting Information file with our resubmission.

4. **Financial Disclosure:** We have updated our financial disclosure as requested. The statement now reads: "GS and JS - Swiss National Science Foundation (SNSF) grant number 200021 207430. The funders had no role in study design, data collection and analysis, decision to publish, or preparation of the manuscript."

5. **Ethics Statement:** The full ethics statement has now been integrated into the manuscript at the end of the Literature Review section (Section 2, page 6). The statement includes the full name of the approving committee ("Ethics Committee of the University of Monash") and the relevant project details ("Project Title: Exploring Navigation Skills using different methods, Project ID: 23126").

6. **Copyrighted Map Images:** We have addressed the issue of potentially copyrighted map images in the original Figures 1 and 4. The revised manuscript now only includes new, fully compliant illustrations. Therefore, we opted to remove Figure 1.
7. **Figure 3 Reference:** We have carefully checked all in-text figure references throughout the manuscript. All placeholder references (e.g., "???") have been replaced with the correct figure numbers, and we confirm that Figure 3 is now correctly referenced in the text.
8. **Supporting Information Captions:** Captions for all Supporting Information files have been added at the end of the manuscript, as per the journal's guidelines.
9. **Identifying Data in Supporting Information:** We have reviewed all Supporting Information files to ensure participant privacy. All potentially identifying data has been removed or fully anonymised. Please let us know if there is anything critical in the data set.
10. **Consent for Interview Transcripts:** We would like to clarify that our research did not involve the collection of interview transcripts. Study 3 was a design studio workshop, not a series of interviews. In accordance with our ethics approval, all participants provided written informed consent for their contributions (including anonymised quotes and design sketches) to be used for research purposes and publication.

Reviewer 1

We are grateful to Reviewer #1 for their valuable and constructive feedback. These comments have significantly improved the manuscript's structure, clarity, and engagement with the current state of the literature. Below, we address each point in detail.

1. **References:** We agree with the reviewer that the literature review needed updating. We have substantially revised the Literature Review (Section 2) to incorporate more recent and relevant studies, ensuring the manuscript reflects the current state of the field. As examples, we have added new citations such as Dugas et al. [5] and Mazurkiewicz et al. [6] that discuss contemporary challenges in pedestrian navigation.
2. **Submission Readiness:** We apologise for the formatting errors in the initial submission.
3. **Unsupported Claims:** We thank the reviewer for highlighting areas where our claims required stronger support.

◦ **3a. (Page 2 Claim)** The claim that turn-by-turn (TBT) instructions are not the best navigation option for pedestrians has now been substantiated with multiple citations. In the revised manuscript (page 2), this statement is now supported by references [1, 5, 6, 12, 13].
◦ **3b. (Page 3 Claim)** The statement regarding the route previews (RP) potential to outperform TBT has been rephrased to be more nuanced. It is now presented as a potential avenue for future design improvements that our study explores, rather than an unsubstantiated claim (page 3).
◦ **3c. (Contradictory Claims)** We have revised the text to remove any contradictions. The summary in Section 3.2.4 (page 9) now accurately reflects the quantitative data presented in the results (a 43.7% preference for RP), stating that a "considerable share" of participants preferred RP, which avoids any overstatement.

4. **Article Structure in Introduction:** A new paragraph has been added at the end of the Introduction (page 3) that explicitly outlines the structure of the article, guiding the reader through the subsequent sections.
5. **Section 2 Title:** The title of Section 2 has been changed from "Related Works" to "Literature Review" to reflect its content more accurately. The introductory text of the section has also been revised accordingly.
6. **Survey Number in Table Captions:** We have updated all relevant table captions to include the corresponding study number for improved clarity (e.g., Table 1, Table 2, and Table 3).
7. **Small Sample Size in Study 3:** We acknowledge the reviewer's concern about the small sample size in Study 3. The Introduction to Study 3 (Section 5, page 15) has been revised to better clarify its qualitative, exploratory nature as a design studio workshop. Furthermore, we have added a paragraph to the Discussion (page 20) that explicitly acknowledges the implications of this small sample size as a limitation of the study.

We believe these changes have greatly strengthened the manuscript and thank the reviewer again for their guidance.

Reviewer 2

We are particularly grateful to Reviewer #2 for their incisive critique. These comments challenged us to re-evaluate the manuscript's core narrative, prompting a significant reframing that has substantially elevated its scientific contribution

1. **Lack of Clear Research Questions:** We agree that research questions help to better guide readers. We have formulated three explicit Research Questions (RQ1, RQ2, RQ3), which are now presented in the Introduction (page 2). These questions frame the entire study by clarifying the specific literature gaps we aim to address concerning pedestrian preferences, objective performance, and design enhancements for navigation aids.
2. **Justification for Three Studies:** The newly introduced Research Questions provide a clear and logical justification for the three-stage study design. The manuscript now explicitly links each study to a specific research question, creating a cohesive narrative:
   ◦ **Study 1** addresses **RQ1** by investigating the *stated preferences* of pedestrians.
   ◦ **Study 2** addresses **RQ2** by evaluating the *objective performance* of the two navigation modes.
   ◦ **Study 3** addresses **RQ3** by exploring potential *design enhancements* for RP.
3. **Flawed Design of Study #1:** We acknowledge the inherent limitations of using online surveys for assessing contextual preferences. In the revised manuscript, we have added a new paragraph in Section 3 (page 6) that explicitly recognises this limitation. The text now frames

Study 1 as an essential first step that provides a baseline understanding of user attitudes, which is then tested against observed behaviour in Study 2.
4. **Flawed Design of Study #2:** We have added a comprehensive new paragraph in the methodology section for Study 2 (Section 4.1.1, page 10) to justify our design choices more robustly. This section now explains:
◦ The rationale for comparing TBT and RP on spatial learning tasks, which reflects a realistic user need to recall routes even when using a navigation aid.
◦ The controls we implemented to manage participant familiarity with the environment. This includes the recruitment of newly enrolled students and the use of familiarity as a covariate in our regression analysis, which confirmed its expected effect (Table 3).
◦ How these additions provide a more rigorous and balanced methodological framework for the field study, strengthening the validity of our findings. These additions not only resolve the reviewer's specific methodological concerns but also provide a more rigorous framework that strengthens our central—and perhaps surprising—finding: that a feature-limited RP mode performs on par with a fully-featured TBT system, even under more stringently controlled conditions.
5. **Argument for Study #3:** The Introduction to Study 3 (Section 5, page 15) has been completely rewritten. It is now clearly framed as the logical next step that directly follows from the findings of Studies 1 and 2. It addresses the key design question that emerges from our results: *how could RP be improved to outperform TBT potentially?*
We are confident that these substantial revisions have addressed the reviewer's critical concerns and have resulted in a significantly stronger and more coherent manuscript. Thank you once again for your time and effort.

Reviewer 3

We thank Reviewer #3 for their careful reading and helpful suggestions, which have improved the manuscript's professionalism and readability.
1. **Date Formatting:** We have corrected the date formatting in Section 5.1. The date is now spelt out as "June 12, 2024" (page 15) to prevent any ambiguity.
2. **Figure Link Placeholders:** The entire manuscript has been reviewed, and all placeholder figure references have been corrected with the proper figure numbers.
3. **Citation Formatting:** The reference list has been carefully checked, and the formatting of all citations has been standardised for consistency throughout the list.

Conclusion

We once again thank the editor and all reviewers for the time and effort invested in providing this constructive feedback. We are confident that the extensively revised manuscript is now a much stronger scientific contribution and is suitable for publication in *PLOS ONE*. We look forward to your evaluation of the revised submission.
Kind regards,

Gian-Luca Savino, Emanuel de Bellis, Reuben Kirkham, Johannes Schöning (Corresponding Author)
